# Coronavírus e o Coração | Um Relato de Caso sobre a Evolução da COVID-19 Associado à Evolução Cardiológica

**DOI:** 10.36660/abc.20200263

**Published:** 2020-05-22

**Authors:** Arthur Rente, Delcio Uezato, Karina Margareth Kunyoshi Uezato

**Affiliations:** 1 Hospital Municipal de Emergências Albert Sabin São CaetanoSP Brasil Hospital Municipal de Emergências Albert Sabin – Emergência,São Caetano, SP – Brasil; 2 Beneficência Portuguesa de São Caetano do Sul São CaetanoSP Brasil Beneficência Portuguesa de São Caetano do Sul – Cardiologia,São Caetano, SP – Brasil

**Keywords:** Doenças Cardiovasculares, Coronavirus, COVID 19, Miocardite/complicações, Insuficiência Respiratória

## Introdução

Com início em 31 de dezembro de 2019, na China, o coronavírus (Sars-Cov-2)^1^ tem sido alvo de estudos das mais diversas áreas médicas, tendo a cardiologia como um dos grandes pilares de evolução.

Doenças crônicas como hipertensão arterial, diabetes melito e doença arterial coronariana aumentam drasticamente o desfecho negativo de pacientes infectados. De acordo com dados da American College of Cardiology (ACC), nesse perfil da população em geral, os níveis de hospitalizações decorrentes de COVID-19 chegam a 50%.^2^

Em virtude de alterações infecciosas decorrentes de infecção, comorbidades crônicas que até então estavam estabilizadas podem tender a descompensação devido à alteração de oferta e demanda de O_2_ , dentre outros fatores de respostas fisiológicas frente a quadros sépticos.^3^

Sabe-se previamente que em outras pandemias de etiologia virais, tais como SARS e MERS, cursaram com quadros de miocardite aguda com desfecho trágico do ponto de vista cardiológico, ficando assim a possiblidade de termos a COVID-19 como fonte de miocardite aguda.^4^

Conforme dados da Sociedade Brasileira de Cardiologia (SBC), em março de 2020, o acometimento cardiovascular relacionado ao novo coronavírus apresentou como desfecho arritmias (16%), isquemia miocárdica (10%), miocardite (7,2%) e choque (1-2%).^5^

## Objetivo

Relato de caso de um paciente portador de diabetes melito que contraiu de forma comunitária o novo coronavírus, evoluiu com alterações cardíacas e foi a óbito.

## Métodos

As informações contidas nesta descrição de caso clínico foram obtidas por meio de revisão de prontuário, entrevista com equipe médica, registro de imagens de exames diagnósticos e revisão de literatura.

## Relato de Caso

Paciente R.S.C., 33 anos de idade, procurou serviço de emergência em hospital municipal de emergências de São Caetano do Sul, estado de São Paulo, em 12 de março de 2020, com quadro de febre não aferida, dor no corpo e tosse produtiva há 3 dias. Passou por avaliação pelo plantonista do pronto-socorro, em que apresentou exame físico sem alterações descritas, foi medicado com sintomáticos e apresentou melhora, sendo liberado após.

**Figura 1:**
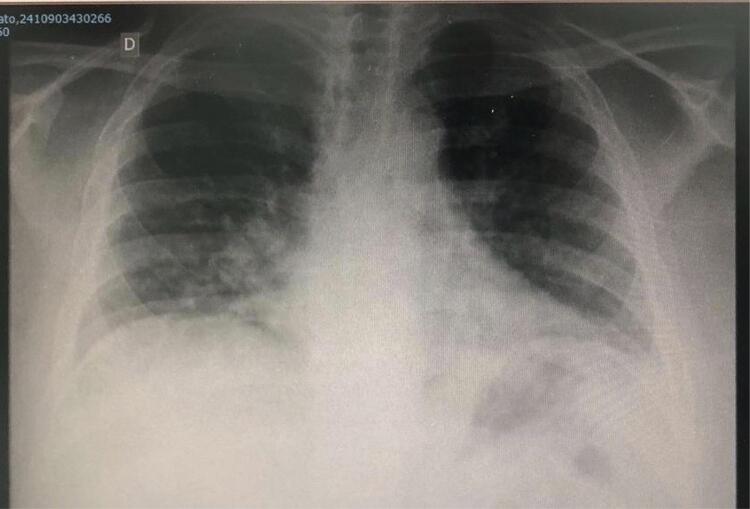
– *Radiografia de tórax em primeiro atendimento.*

Em 14 de março de 2020, o paciente retornou ao pronto atendimento referindo tosse seca, sensação de dispneia e febre (39 graus – aferido em madrugada prévia). Negou congestão ou rinorreia. Foi realizada radiografia de tórax ( Figura 1 ) e a prescrição médica foi amoxicilina + clavulanato 875/125mg, de 12 em 12 horas. O paciente foi liberado posteriormente.

Em 16 de março de 2020, o paciente retornou, com piora de sensação de dispneia, sem melhora de adinamia e aumento de tosse seca; febre havia cessado, mas apresentava sudorese intensa. Ao exame físico, evoluía com diminuição de murmúrio vesicular bilateralmente.

**Figura 2:**
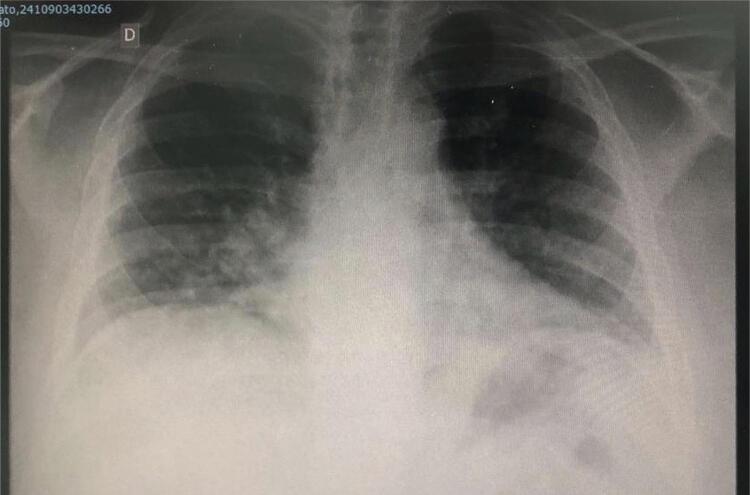
– *Radiografia de tórax referente ao segundo atendimento*

Foi realizada nova radiografia de tórax ( Figura 2 ), em que foi apresentada intensa piora, com radiopacidade pronunciada em campos pulmonares bilaterais, sendo instalada suplementação de O_2_ por cateter nasal, com discreta melhora.

A equipe do pronto atendimento solicitou vaga em enfermaria para elucidação diagnóstica e tratamento.

Em 16 de março de 2020, o paciente foi recebido na enfermaria do hospital de emergências, sendo realizada anamnese de paciente (descrita a seguir).

Paciente admitido em enfermaria com quadro de tosse produtiva, febre há 4 dias, tendo usado amoxicilina + clavulanato por 2 dias sem melhoras; nega viagem para exterior ou contato com pessoa com sintomas de COVID-19.

Tem diabetes de antecedentes pessoais, porém não faz tratamento regular.

Em admissão em enfermaria, paciente apresentava-se em, Glasgow 15, eupneico em cateter de O_2_, acianótico. Ritmo cardíaco regular em 2 tempos; frequência cardíaca: 90 bpm; pressão arterial: 120 x 70mmHg. Murmúrio vesicular presente com roncos difusos esparsos e diminuído globalmente. Radiografia de tórax, radiopacidade pronunciada nos campos pulmonares bilateralmente.

Exames laboratoriais em 16 de março de 2020: Hb: 15,5; leucócitos: 16.150 mil; bastões: 323; Vhs: 23; Na: 135; K: 4,3; ureia: 2; creatinina: 0,6; PCR: 23,6; glicemia: 344.

Aventada hipótese diagnóstica de pneumonia e diabetes descompensada.

Solicitação de tomografia de tórax, isolamento respiratório, pesquisa COVID-19 ( *swab* e PCR).

Optou-se por iniciar piperacilina + tazobactan 4,5 mg, 6/6 horas, correção de dextro.

Em 17 de março de 2020, paciente evoluiu com mal-estar geral, quadro de febre (38,3°C), refratária à medicação; dessaturação 85% em O_2_ 3L/min; taquicardíaco, 104 bpm; taquipneico; 84 ipm; pressão arterial: 120x70mmHg.

Ritmo cardíaco taquicardíaco com abafamento de bulhas, dor torácica, turgência jugular com sinais de disfunção diastólica.

Murmúrio vesicular presente com estertores crepitantes por todo hemitórax direito.

Em 17 de março de 2020, eletrocardiograma não apresentou sinais de isquemia.

Exames laboratoriais em 17 de março de 2020: Hb: 14,2; hT: 41%; leucocitose: 15.030; plaquetas: 143 mil; PCR: 25; ureia: 36; creatinina: 0,60; Na: 135; K: 4,2.

Formulada hipótese de sepse de foco pulmonar; etiologia viral, equipe optou por obtenção de via aérea definitiva com intubação orotraqueal.

Pressão arterial: 154x91 mmHg; FC: 48; bpm sat O_2_: 82%; FiO_2_: 100%; Peep: 12; pressão controlada.

**Figuras 3 a 6:**
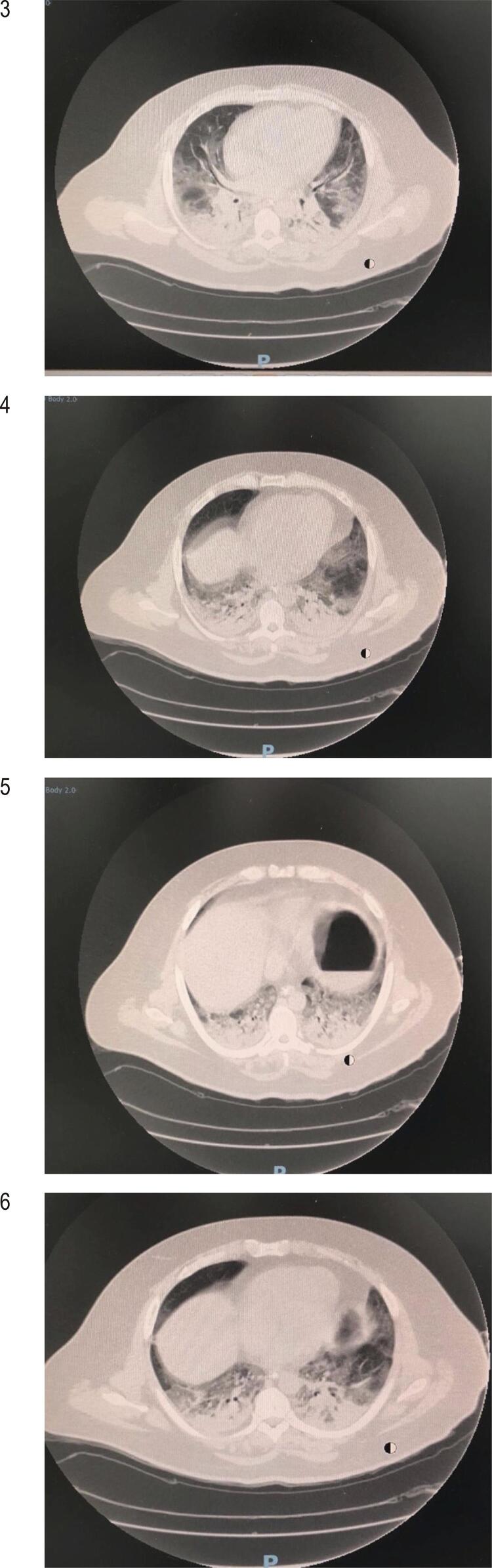
– *Tomografia de tórax em janela pulmonar com acometimento padrão vidro fosco.*

Devido à instabilidade hemodinâmica, o paciente foi mantido em sala de emergência à espera de vaga na unidade de terapia intensiva (UTI). Foi realizada tomografia computadorizada (TC) de tórax ( Figuras 3 a 6 ), que apresentava intubação seletiva de brônquio fonte direito; opacidades em vidro fosco extensas em ambos os pulmões, comprometendo todos os lobos com predomínio em lobos inferiores, em que se notam consolidações com broncogramas aéreos, o que sugere processo inflamatório/infeccioso. Não é possível afastar etiologia viral. Há presença de edema miocárdico secundário a processo inflamatório associado a espessamento de parede miocárdica com discreto aumento de área cardíaca.

Realizada nova intubação orotraqueal com sucesso, mantidas antibioticoterapia e medidas para insuficiência respiratória aguda.

Em 18 de março de 2020, o paciente evoluiu para mal-estar geral – apresentou-se taquicardíaco, taquidispneico sedado (Ramsey 6) em ventilação mecânica.

Ritmo cardíaco taquicardíaco, com hipofonese de b1 e b3 em esboço, presença de turgência jugular, edema MMII 1/4+.

Murmúrio vesicular com roncos difusos.

Iniciada observação para miocardite aguda + sepse de foco pulmonar.

Mantida antibioticoterapia associada a monitoramento hemodinâmico; início de fármaco vasoativo caso necessário.

Vaga de UTI para paciente disponibilizada em hospital das clínicas (HC) de São Paulo, porém paciente evoluiu para óbito no momento de admissão devido a quadro de insuficiência respiratória aguda – COVID-19. Resultados relevantes:

Em 17 de março de 2020, foram realizados testes complementares que apresentaram os seguintes resultados:

- Troponina quantitativa: 0,49 ng/dL
- Dímero D: 0,1 mg/L
- Lactato venoso: 2,0 mmol/L
- Teste rápido HIV: negativo

Foi realizado *swab* orofaríngeo (teste rápido para COVID-19) em 17 de março de 2020, com resultado negativo; no entanto, é notório que a especificidade de *swab* nasal gira em torno de 63%, sendo utilizado em primeira triagem.

O exame padrão-ouro para detecção de COVID-19 consiste em coleta de amostra *in vivo* ou *post mortem* de PCR (PCR 2019-nCoV), em que foi obtido o resultado positivado da amostra (coleta em 19 de março de 2020 – *post mortem* ). Os exames realizados no estado de São Paulo – obtidos por meio do Sistema Único de Saúde (SUS) – foram analisados pelo Instituto Adolfo Lutz Central, concluindo o diagnóstico de infecção pelo novo coronavírus.

## Discussão

Tendo em vista a atual pandemia e uma doença ainda em investigação, não podemos descartar o acometimento em jovens e crianças, principalmente naqueles portadores de agravos crônicos. Há relatos de vivência clínica que demonstram que muitos pacientes entre 20 a 40 anos de idade estão sendo infectados pelo novo coronavírus, desenvolvendo múltiplas comorbidades associadas à prevalência infecciosa.

O acometimento cardíaco, que leva a quadros de insuficiência cardíaca aguda, vem sendo apontando como uma das maiores fontes de complicações secundárias, com desfecho reservado, sem uma terapia especifica para tratamento, somente o seguimento clássico de insuficiência cardíaca aguda já preconizado em nossas diretrizes, além de controle do foco infeccioso.

## Conclusão

O desfecho cardiovascular é uma possibilidade real na vivência clínica da pandemia do novo coronavírus, sendo necessários o monitoramento e o acompanhamento de insuficiência cardíaca aguda.^6^

Os sinais clínicos devem sempre nos guiar a aventar tais possibilidades, além de mantermos o alerta para miocardites.

Exames complementares como TC e radiografia de tórax são úteis para investigação, e o ecocardiograma pode facilitar o manejo e a conduta. Vale lembrar que, muitas vezes, não há disponibilidade em unidades de pronto atendimento, e também apresentar resultados variáveis por ser um exame com resultado operador dependente.

Em virtude da gravidade dos pacientes (a maioria sob ventilação mecânica), a ressonância cardíaca não apresenta grande valia devido à impossibilidade de ser realizada.

Corroboramos a necessidade de anamnese e de avaliação clínica/cardiológica minuciosa de tal perfil de paciente, a fim de minimizar as evoluções desfavoráveis.
