# Supplementary material for: Coronavírus e o Coração | Um Relato de Caso sobre a Evolução da COVID-19 Associado à Evolução Cardiológica
Source: Arq Bras Cardiol. 2020 May 22;114(5):839–42. [Article in Portuguese] doi: 10.36660/abc.20200263 (PMC8387009; doi:10.36660/abc.20200263)
Supplement: Translation

# Translation: Coronavirus and the Heart | A Case Report on the Evolution of COVID-19 Associated with Cardiological Evolution

**Authors:** Arthur Rente, Delcio Uezato, Karina Margareth Kunyoshi Uezato

**Affiliations:** 1 Brazil Hospital Municipal de Emergências Albert Sabin – Emergência, São Caetano, SP – Brazil; 2 Brazil Beneficência Portuguesa de São Caetano do Sul – Cardiologia, São Caetano, SP – Brazil

**Keywords:** Cardiovascular Diseases, Coronavirus, COVID 19, Myocarditis/complications, Respiratory Insufficiency

## Introduction

Beginning on December 31, 2019, in China, the coronavirus (Sars-Cov-2)^1^ has been the target of studies in the most diverse medical fields, with cardiology as one of the major pillars of evolution.

Chronic diseases such as hypertension, diabetes mellitus and coronary artery disease dramatically increase the negative outcome of infected patients. According to data from the American College of Cardiology (ACC), in this profile of the general population, hospitalization levels resulting from COVID-19 hit 50%^2^

Due to infectious abnormalities resulting from infection, chronic comorbidities that until then were stabilized may tend to decompensate due to changes in O_2_supply and demand, among other factors of physiological responses to septic conditions.^3^

It is known that, previously, other pandemics of viral etiology, such as SARS and MERS, involved acute myocarditis with a tragic outcome from the cardiological point of view, and COVID-19 may be a source of acute myocarditis.^4^

According to data from the Brazilian Society of Cardiology (SBC), in March 2020, cardiovascular impairment related to the novel coronavirus has the following outcomes: arrhythmias (16%), myocardial ischemia (10%), myocarditis (7.2%) and shock (1–2%).^5^

## Objective

Case report of a patient with diabetes mellitus who contracted the novel coronavirus in community, evolved with cardiac disorders and died.

## Methods

The information included in this clinical case description derived from reviews of medical records, interviews with the medical team, diagnostic imaging tests and literature reviews.

## Case Report

R.S.C., 33 years old, sought the emergency service at a municipal emergency hospital in São Caetano do Sul, state of São Paulo, on March 12, 2020, with unverified fever, body pain and wet cough for 3 days. The patient was evaluated by the emergency duty manager, where he was physically examined but presented no abnormalities, was medicated, presented symptoms, showed improvement and was discharged.

On March 14, 2020, the patient returned to the emergency room reporting dry cough, dyspnea and fever (39 degrees — measured in the early morning). Denied congestion or rhinorrhea. Chest radiography ( Figure 1 ) was performed and the medical prescription was amoxicillin + clavulanate 875/125 mg every 12 hours. The patient was later discharged.

**Figure 1:** – *Chest radiography at first visit.*

On March 16, 2020, the patient returned with worsened sensation of dyspnea, no improvement of adynamia and increased dry cough; the fever had ceased, but the patient presented severe sweating. On physical examination, the patient progressed with decreased bilateral breath sounds.

A new chest X-ray was performed ( Figure 2 ), which presented intense worsening, radiopacity bilaterally pronounced in lung fields, with supplementation of O_2_by a nasal catheter, with slight improvement.

**Figure 2:** – *Chest radiography referring to the second appointment.*

The emergency room team requested a place in the ward to clarify diagnosis and treatment.

On March 16, 2020, the patient was received at the emergency hospital ward and history-taking was performed (as described below).

Patient admitted to the ward with wet cough, fever for 4 days, taking amoxicillin + clavulanate for 2 days without improvement; no travel abroad or contact with anyone with symptoms of COVID-19.

Diabetes with personal history, but not regular treatment.

Admission to the ward. Glasgow 15, eupneic in O_2_catheter, acyanotic. Two-stroke regular heart rhythm; heart rate: 90 bpm; blood pressure: 120 x 70 mmHg. Globally decreased vesicular murmur with sparse diffuse snores. Chest radiography, radiopacity bilaterally pronounced in the lung fields.

Laboratory tests on March 16, 2020: Hb: 15,5; leukocytes: 16,150 mil; band cells: 323; Vhs: 23; Na: 135; K: 4.3; urea: 2; creatinine: 0,6; CRP: 23,6; blood glucose: 344.

Diagnostic hypothesis of pneumonia and decompensated diabetes was suggested.

Request for chest tomography, respiratory isolation, COVID-19 investigation (swab and CRP).

Piperacillin + tazobactan 4.5 mg every 6 hours was initiated, dextro-transposition.

On March 17, 2020, the patient evolved with general malaise, fever (38.3 °C), refractory to medication; 85% desaturation in O_2_3L/min; tachycardia, 104 bpm; tachypneic; 84 ipm; blood pressure: 120x70 mmHg.

Tachycardiac heart rhythm with muffled heart sounds, chest pain, jugular venous distension with signs of diastolic dysfunction.

Vesicular murmur with crackles all across the right hemithorax.

On March 17, 2020, electrocardiography presented no signs of ischemia.

Laboratory tests on March 17, 2020: Hb: 14,2; hT: 41%; leukocytosis: 15.030; platelets: 143 mil; CRP: 25; urea: 36; creatinine: 0.60; Na: 135; K: 4.2.

Hypothesis of pulmonary focus sepsis; viral etiology. The patient opted for a definitive airway with orotracheal intubation.

Blood pressure: 154x91 mmHg; HR: 48; bpm sat O_2_: 82%; FiO_2_: 100%; Peep: 12 bpm; controlled blood pressure:

Due to hemodynamic instability, the patient was kept in the emergency room waiting for a place in the intensive care unit (ICU). Computed tomography (CT) of the chest ( Figures 3 to 6 ) revealed selective intubation of the right mainstem bronchus; extensive ground-glass opacity in both lungs, affecting all lobes predominantly in the lower lobes, which present consolidations with air bronchograms, suggesting inflammation/infection. Viral etiology cannot be ruled out. Presence of myocardial edema secondary to an inflammatory process associated with myocardial wall thickening with a slight increase in the cardiac area.

**Figures 3 to 6:** – *Chest tomography in pulmonary window with standard ground-glass involvement.*

New successful orotracheal intubation, antibiotic therapy and measures for acute respiratory failure maintained.

On March 18, 2020, the patient developed general malaise — tachycardia, sedated, tachydyspneic (Ramsey 6), on mechanical ventilation.

Tachycardiac cardiac rhythm, with hypophonesis of b1 and b3 in outline, presence of jugular venous distension, edema on lower limbs 1/4+.

Vesicular murmur with diffuse snores.

Observation for acute myocarditis + pulmonary focus sepsis was initiated.

Antibiotic therapy associated with hemodynamic monitoring was maintained if necessary; initiation of vasoactive drug.

ICU spot was provided at Hospital das Clínicas (HC) de São Paulo, but the patient died on admission due to respiratory failure — COVID-19.

Relevant findings:

On March 17, 2020, additional tests presented the following findings:

- Quantitative troponin: 0.49 ng/dl
- D-dimer: 0.1 mg/L
- Venous lactate: 2.0 mmol/L
- Rapid HIV test: negative

Oropharyngeal swab (rapid test for COVID-19) was performed on March 17, 2020 and tested negative; however, it is clear that the specificity of nasal swab is around 63%, so it is used in the first screening.

The gold standard test for detecting COVID-19 consists of collecting in vivo or post-mortem CRP samples (2019-nCoV CRP), which tested positive (collected on March 19, 2020 — post-mortem). The tests performed in the state of São Paulo — sourced from the Brazilian public health system (SUS) — were analyzed by Instituto Adolfo Lutz Central, completing diagnosis of infection with the novel coronavirus.

## Discussion

In view of the current pandemic and a disease still under investigation, we cannot rule out infection among young people and children, especially those with chronic conditions. Reports of clinical experience demonstrate that many patients aged 20 to 40 are being infected by the novel coronavirus, developing multiple comorbidities associated with the infection.

Cardiac involvement, which leads to acute heart failure, has been identified as one of the major sources of secondary complications, with reserved outcome, without specific therapy, except for the classic follow-up of acute heart failure as recommended in our guidelines, in addition to control of the focus of infection.

## Conclusion

The cardiovascular outcome is a real possibility in the clinical experience of the novel coronavirus pandemic, requiring monitoring and follow-up of acute heart failure.^6^

Clinical signs should always guide us to consider these possibilities, in addition to keeping an eye for myocarditis.

Complementary tests such as CT and chest radiography are useful for investigation, and echocardiography can facilitate management. It is worth noting that it is not often available in emergency care units in order to have its operator-dependent results.

Due to the severity of the patients’ conditions (mostly under mechanical ventilation), cardiac resonance imaging is not of great value as it cannot be performed.^6^

We corroborate the need for history-taking and thorough clinical/cardiological evaluation of such patient profile in order to minimize unfavorable outcomes.
